# Are graph databases ready for bioinformatics?

**DOI:** 10.1093/bioinformatics/btt549

**Published:** 2013-10-17

**Authors:** Christian Theil Have, Lars Juhl Jensen

**Affiliations:** ^1^Department of Metabolic Genetics, Novo Nordisk Foundation Center for Basic Metabolic Research, University of Copenhagen, 2100 Copenhagen Ø, Denmark and ^2^Department of Disease Systems Biology, Novo Nordisk Foundation Center for Protein Research, University of Copenhagen, 2100 Copenhagen N, Denmark

## Abstract

**Contact:** Lars.Juhl.Jensen@gmail.com

Graphs are ubiquitous in bioinformatics and frequently consist of too many nodes and edges to represent in random access memory. These graphs are thus stored in databases to allow for efficient queries using declarative query languages such as Structured Query Language (SQL). Traditional relational databases (e.g. MySQL and PostgreSQL) have long been used for this purpose and are based on decades of research into query optimization. Recently, NoSQL databases have caught a lot of attention because of their advantages in scalability. The term NoSQL is used to refer to schemaless databases such as key/value stores (e.g. Apache Cassandra), document stores (e.g. MongoDB) and graph databases (e.g. AllegroGraph, Neo4J, OpenLink Virtuoso), which do not fit within the traditional relational paradigm. Most NoSQL databases do not have a declarative query language. The widely used Neo4J graph database is an exception (Webber *et al.*, 2013). Its query language Cypher is designed for expressing graph queries, but is still evolving.

Graph databases have so far seen only limited use within bioinformatics (Schriml *et al.*, 2012). To illustrate the pros and cons of using a graph database (exemplified by Neo4J v1.8.1) instead of a relational database (PostgreSQL v9.1), we imported into both the human interaction network from STRING v9.05 (Franceschini *et al.*, 2013), which is an approximately scale-free network with 20 140 proteins and 2.2 million interactions. As all graph databases, Neo4J stores edges as direct pointers between nodes, which can thus be traversed in constant time. Because Neo4j uses the property graph model, nodes and edges can have properties associated with them; we use this for storing the protein names and the confidence scores associated with the interactions (Fig. 1). In PostgreSQL, we stored the graph as an indexed table of node pairs, which can be traversed with either logarithmic or constant look up complexity depending on the type of index used. On these databases we benchmarked the speed of Cypher and SQL queries for solving three bioinformatics graph processing problems: finding immediate neighbors and their interactions, finding the best scoring path between two proteins and finding the shortest path between them. We have selected these three tasks because they illustrate well the strengths and weaknesses of graph databases compared with traditional relational databases.

A common task in STRING is to retrieve a neighbor network. This involves finding the immediate neighbors of a protein and all interactions between them. To express this as a single SQL query requires the use of query nesting and a UNION set operation. Because Cypher currently supports neither of these features, two queries are needed to solve the task: one to find immediate neighbors and a second to find their interactions, which must be run for each of the immediate neighbors. Although this precludes some query optimizations, running all these Cypher queries is 36× faster than running the single SQL query (Table 1). However, it should be noted that a 49× fold speedup is attainable with PostgreSQL by similarly decomposing the complex query into multiple simple SQL queries. In theory, posing the task as one declarative query maximizes the opportunity for query optimization, but in practice this does not always give good performance. These results also show that even for graph data, using a graph database is not necessarily an advantage.

**Table 1.** Query benchmark of a relational and a graph database

	Neighbor network	Best-scoring path	Shortest path
PostgreSQL	206.31 s	1147.74 s	976.22 s
Neo4j	5.68 s^a^	1.17 s	0.40 s
Speedup	36×	981×	2441×

*Note*: For each of three selected tasks, we ran the corresponding queries for randomly selected human proteins/protein pairs and report the average time. We used a Linux machine equipped with a 3GHz quad-core Intel Core i3 processor, 4 GB random access memory and a 250 GB 7200 rpm hard drive.

^a^Neighbor networks cannot be expressed as a single Cypher query. Instead we report the total time of all queries involved in solving this task. Similar speedup was observed for PostgreSQL when similarly decomposing the complex query into multiple simple queries.

Finding the best scoring path in a weighted graph is another frequently occurring task. For example, finding the best scoring path connecting two proteins in the STRING network is a crucial part of the NetworKIN algorithm (Linding *et al.*, 2007). This task can be expressed single query both in (recursive) SQL and in Cypher. However, in practice neither query can be executed unless the maximal path length is severely constrained, in which case the Cypher query was faster by a factor of 981× (Table 1). The poor scalability is because of an exponential explosion in the number of longer paths, which in part is because of the scale-free nature of the network. The task can be efficiently solved using Dijkstra’s algorithm, but neither database is capable of casting queries as dynamic programming problems, although promising results have been achieved with automatic dynamic programming in declarative languages (Zhou *et al.*, 2010).

By contrast, the Cypher graph query language has a dedicated function for finding shortest paths, not taking into account edge weights. This leads to a massive speed improvement for this specific task: Neo4j is able to find the shortest path with no length constraint 2441× faster than PostgreSQL can find the shortest path when constraining the maximal path length to two edges. This shows what is possible when tightly integrating efficient algorithms with graph databases.

In summary, graph databases themselves are ready for bioinformatics and can offer great speedups over relational databases on selected problems. The fact that a certain dataset is a graph, however, does not necessarily imply that a graph database is the best choice; it depends on the exact types of queries that need to be performed. Graph queries formulated in terms of paths can be concise and intuitive compared with equivalent SQL queries complicated by joins. Nevertheless, declarative graph query languages leave much to be desired, both feature-wise and performance-wise. Relational databases are a better choice when set operations are needed. Such operations are not as natural a fit to graph databases and have yet to make it into declarative graph database query languages. These languages are efficient for basic path traversal problems, but to realize the full benefits of using a graph database, it is presently necessary to tightly integrate the relevant algorithms with the graph database.

**Fig. 1.**
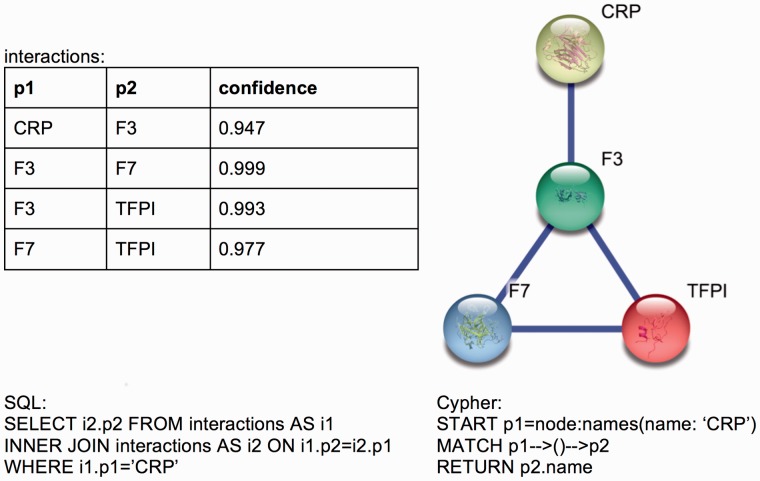
Relational versus graph database representation of a small protein interaction network. In the relational database, the network is stored as an interactions table (left). By contrast a graph database directly stores interactions as pointers between protein nodes (right). Below, we show the queries to identify second-order interaction partners in SQL and Cypher, respectively

*Conflict of Interest*: none declared.
